# Comparative analysis of the clinical effects of different thoracoscopic resection in the treatment of Stage I Non-Small Cell Lung Cancer

**DOI:** 10.12669/pjms.40.8.9124

**Published:** 2024-09

**Authors:** Hao Jiang, Tong Wu, Peng Qie, Huien Wang, Baoxin Zhang

**Affiliations:** 1 Hao Jiang Department of Thoracic Surgery, Cangzhou People’s Hospital, Cangzhou 061000, Hebei, China; 2 Tong Wu Department of Thoracic Surgery, Cangzhou People’s Hospital, Cangzhou 061000, Hebei, China; 3 Peng Qie Department of Thoracic Surgery, Hebei Provincial People’s Hospital, Shijiazhuang 050051, Hebei, China; 4 Huien Wang Department of Thoracic Surgery, Hebei Provincial People’s Hospital, Shijiazhuang 050051, Hebei, China; 5 Baoxin Zhang Department of Thoracic Surgery, Cangzhou Hospital of Integrated Traditional Chinese and Western, Medicine of Hebei Province, Cangzhou 061000, Hebei, China

**Keywords:** Thoracoscopy, Lobectomy, Segmental pneumonectomy, Stage I, Non-small cell lung cancer, Pulmonary function, Prognosis

## Abstract

**Objective:**

To compare and analyze the clinical effects of thoracoscopic lobectomy and segmentectomy in stage I non-small cell lung cancer (NSCLC).

**Method:**

This was a retrospective study. Eighty patients with stage I NSCLC treated in Cangzhou People’s Hospital from December 2019 to January 2022 were randomly divided into the segmentectomy group and lobectomy group, with 40 cases in each group. Further comparative analysis was carried out focusing on perioperative indexes, maximum ventilation volume (MVV), forced vital capacity (FVC), forced expiratory volume in the first second (FEV1), VAS score of postoperative pain and complications.

**Result:**

There was no significant difference in the number of dissected lymph nodes and extubation time between the two groups (p>0.05). The operation time was longer, while intraoperative blood loss was less and the stay of stay in hospital was shorter in the segmentectomy group significantly than those in the lobectomy group (p<0.05). Furthermore, no significant difference was observed in MVV%, FVC% and FEV1% between the two groups before operation (p>0.05). Meanwhile, the segmentectomy group had evidently lower VAS scores at 1 d, 3 d and 5 d postoperatively than those in the lobectomy group (p<0.05). Besides, there was a much lower total incidence of complications in the segmentectomy group than that in the lobectomy group (p<0.05).

**Conclusion:**

Compared with lobectomy, thoracoscopic segmentectomy is more effective in the treatment of stage I NSCLC, with less bleeding and mild pain, which can alleviate pulmonary function injury and reduce postoperative complications that is conducive to the improved prognosis of patients.

## INTRODUCTION

Lung cancer is the leading cause of morbidity and mortality of malignant tumors, posing a serious threat to the life and safety of patients.^1^,^2^ Non-small cell lung cancer(NSCLC) comprises the vast majority (about 80%~85%)^3^,^4^ of lung cancer, presented with clinical symptoms such as chest tightness, chest pain, cough, hemoptysis, etc. The development of endoscopy and imaging techniques have promoted public awareness of lung cancer prevention and control gradually, leading to an increase in the diagnosis of early NSCLC. Stage I NSCLC is characterized by a relatively small range of lesions, no long-term metastasis and no invasion to important thoracic organs.

Therefore, timely surgical treatment can improve the prognosis and survival of these patients.^5^ Thoracoscopic lobectomy is a common surgical choice for the treatment of NSCLC clinically, with less intraoperative blood loss. However, it has disadvantages of potential adhesion of tissue and blood vessels, and unclear surgical field of vision due to the influence of bleeding points possibly, leading to a higher difficulty in separating the lung lobes that may produce negative impact on the surgical effect. Furthermore, prior research has reported the significant therapeutic effect of thoracoscopic segmentectomy in the treatment of early NSCLC.^6^,^7^ However, there are relatively few studies comparing thoracoscopic lobectomy and segmentectomy in the treatment of stage I NSCLC. Accordingly, the present study was performed to compare and analyze the clinical effects of thoracoscopic lobectomy and segmentectomy in stage I NSCLC patients.

## METHODS

This was a retrospective study. Eighty patients with stage I NSCLC admitted to Cangzhou People’s Hospital from December 2019 to January 2022, and divided into the segmentectomy group and lobectomy group, with 40 cases in each group according to different treatment methods. There was no significant difference in gender, age, tumor diameter, pathological type, tumor location and TNM stage between the two groups(p>0.05), suggesting the comparability between groups, as shown in Table-I.

**Table-I:** Comparison of general data between the two groups (*χ̅*±*S*); (n, %).

Groups	n	Gender		Age (years)	Tumor diameter (cm)	Pathological types			Tumor location		TNM stage	
	
		Male	Female			Adenocarcinoma	Squamous cell carcinoma	Others	Left lung	Right lung	I a	I b
Segmentectomy group	40	18 (45.00)	22 (55.00)	63.52±5.82	1.64±0.43	25 (62.50)	13 (37.50)	2 (5.00)	16 (40.00)	24 (60.00)	26 (65.00)	14 (35.00)
Lobectomy group	40	23 (57.50)	17 (42.50)	63.84±5.73	1.72±0.47	27 (67.50)	10 (25.00)	3 (7.50)	21 (52.50)	19 (47.50)	22 (55.00)	18 (45.00)
*t*/*χ*^2^	-	1.251		-0.248	-0.794	0.668			1.257		0.833	
*P*	-	0.263		0.805	0.429	0.716			0.262		0.361	

### Ethical Approval:

The study was approved by the Institutional Ethics Committee of Cangzhou People’s Hospital (No.: K2021-054; date: May 14, 2021), and written informed consent was obtained from all participants.

### Inclusion criteria:

- Patients with NSCLC diagnosed by postoperative pathological biopsy.
- Patients with TNM clinical stage I.
- Patients with an estimated survival of >six months.
- Patients with tumor diameter <3 cm.
- Patient aged over 50 years old.

### Exclusion criteria:

- Patients who had the previous history of chest surgery or radiotherapy and chemotherapy.
- Patients with surgical contraindications for thoracoscopic segmentectomy or lobectomy.
- Patients with severe systemic infection and malignant tumors of other systems.
- Patients with severe liver and kidney dysfunction.
- Patients lost during follow-up.
- Patients with Complicated asthma, pulmonary edema and other respiratory diseases.

### Methods:

Patients in both groups underwent double-lumen endotracheal intubation, of which lung ventilation was maintained on the healthy side, and collapse was found on the affected side. All patients were fasted for 8 hours and water was forbidden for six hours before operation, under general anesthesia, patients were adjusted to their posture of lying on the healthy side, with a pillow placed under the armpit, the right upper limb lifted up and fixed on the hand bracket. An incision of about 1.5 cm in length (along the 7th intercostal space of the axillary midline) was made as the thoracoscopic observation hole; an incision (about 3.0 cm in length; along the 3rd or 3rd intercostal space of the axillary front line) was made as the main operating hole, and another incision (about 1.5 cm; along the 8th intercostal space of the posterior axillary line) as the auxiliary operating hole, respectively.

### Segmentectomy group:

For thoracoscopic segmentectomy, wedge excision was performed firstly for peripheral pulmonary nodules, and the intraoperative specimens were collected to prepare the frozen section to observe whether the frozen section was completely consistent with the paraffin-embedded section, so as to determine the benign and malignant lesions for subsequent segmentectomy. Segmentectomy would be adopted directly when the nodule was located deeply indicated by CT. A more flexible and precise operation was required when the blood vessels of the lung segment showed multiple variations and were in a deep position in the lung parenchyma. Different surgical procedures were needed in view of the development of different pulmonary fissures in the lung segment.

In case of a good development of the pulmonary fissure in the pulmonary segment, the pulmonary segmental artery was separated firstly in the fissure, and then excised and ligated, followed by the separation of the segmental bronchus into deep parts. The segmental bronchus was temporarily clamped and then cut off. The affected side was ventilated with low pressure and low tidal volume, with close monitoring of the lung inflation. The slowly inflated region of the lung was surgically resected in the pulmonary segment. The segmental bronchus was cut off and partially closed, after which the veins were dissected, the severed segmental veins were ligated, and the predetermined pulmonary segments were removed, followed by routine and systematic lymph node dissection of the mediastinum and hilum.

When there was a poor development of the pulmonary fissure in the pulmonary segment, the lobar vein was exposed firstly, after which the segmental vein was dissected along the lobar vein to the deep part and ligated. Then, the segmental artery and segmental bronchus were dissected along the segmental vein in the pulmonary parenchyma. Subsequently, the target pulmonary segment was excised after the segmental pulmonary artery and segmental bronchus were separated separately. Notably, during resection, the incisal margin should be at least 2 cm away from the tumor. If necessary, the adjacent pulmonary segmental tissue can be removed to ensure a negative margin. Routine dissection was performed for lymph nodes of the mediastinum and hilum.

### Lobectomy group:

Similarly, for thoracoscopic lobectomy, the first step was wedge excision to freeze the collected specimens for section preparation to observe whether the frozen section was completely consistent with the paraffin-embedded section, so as to determine the benign and malignant lesions for subsequent lobectomy. After separation, ligation and cut off of the lobar vein, the lobar bronchus was then dissected, ligated and cut off, so that each branch of the lobar artery can be fully preserved before dissociation. The proximal end was double ligated and cut off with an ultrasonic scalpel. Afterward, he segmental the pulmonary fissure was ligated and cut off, and routine and systematic lymph node dissection was performed in the mediastinum and hilum.

### Observational indexes:

All patients in the two groups were awake within two hours after surgery.

The perioperative indexes of the two groups were compared, including operation time, length of incision, intraoperative blood loss, thoracic drainage, number of lymph node dissected, length of stay in hospital and extubation time;

Pulmonary function was measured by German Siemens ACUSON X300PE color Doppler ultrasonography before operation, one month, three months and six months after operation in the two groups, including maximum ventilation volume (MVV), forced vital capacity (FVC), forced expiratory volume in the first second (FEV1).

The degree of postoperative pain was measured by visual analogue scale (VAS), which were recorded on one day, three da and five days after operation;

The complications of the two groups were compared, including pulmonary infection, pulmonary air leakage, intestinal obstruction,, atelectasis and arrhythmia. All the patients follow-up time was one year.

### Statistical analysis:

Data analysis of this study was realized by using SPSS26.0 software. Counting data were expressed as (n, %) and compared with the χ^2^ test; and measurement data were presented in (*χ̅*±*S*) and processed by independent-sample t test or repeated measurement analysis. Graphpad Prism was employed for the construction of a graphical representation of the data. P<0.05 indicated the existence of a statistically significant difference.

## RESULTS

As shown in Table-II, there was no significant difference in the number of dissected lymph nodes and extubation time between the two groups (p>0.05). The operation time was longer, while intraoperative blood loss was less and the stay of stay in hospital was shorter in the segmentectomy group significantly than those in the lobectomy group (p<0.05).

**Table-II:** Comparison of perioperative indexes between the two groups (*χ̅*±*S*).

Groups	n	Operation time (min)	Intraoperative blood loss (ml)	Thoracic drainage volume (ml)	Number of lymph node dissected (n)	Length of stay in hospital (d)	Extubation time (d)
Segmentectomy group	40	147.77±31.39	136.38±35.71	127.29±29.37	12.22±1.96	7.35±2.11	3.32±0.69
Lobectomy group	40	110.04±27.68	161.09±44.87	153.51±37.43	12.74±2.09	9.75±3.16	3.52±0.78
*t*		5.702	-2.725	0.048	0.595	-3.995	1.216
*P*		<0.001	0.008	0.001	0.255	<0.001	0.228

**Fig.1:**
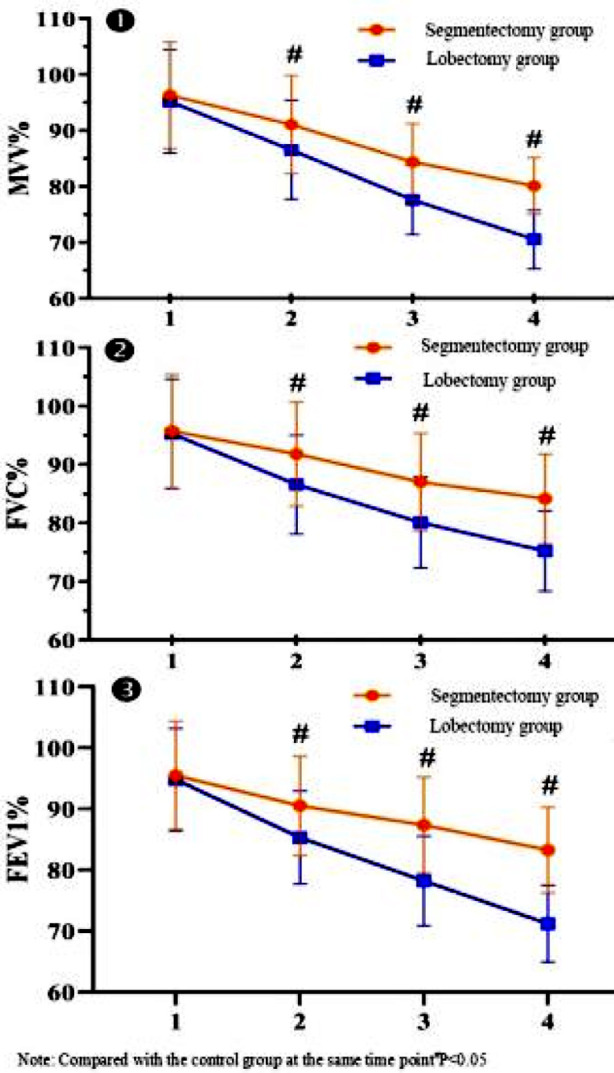
The change trend of pulmonary function indexes in the two groups with time.

**Fig.2:**
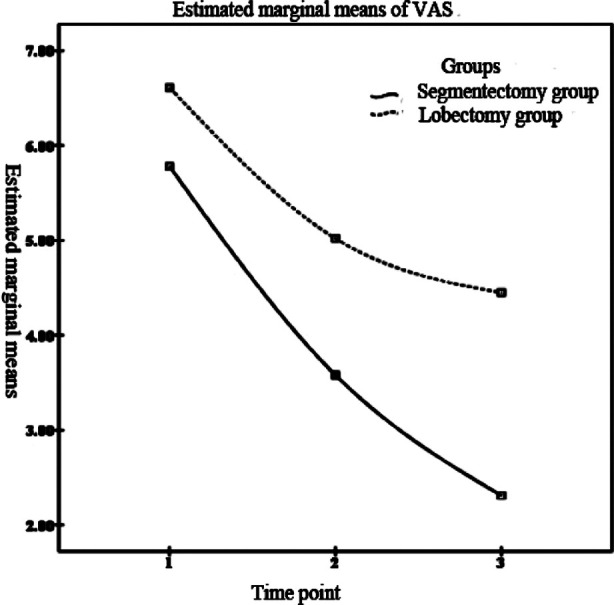
The change trend of VAS score of postoperative pain in the two groups with time.

In terms of pulmonary function, the results of Mauchly’s Test of Sphericity showed that MVV% and FEV1% of the two groups did not meet Huynh-Leldt condition (p<0.05), and FVC% met the condition (p>0.05). Based on repeated measurement analysis, there were significant differences in intra-subject and inter-subject effects of MVV%, FVC% and FEV1% between the two groups (p<0.05). It suggested that MVV%, FVC% and FEV1% changed with time, which varied with different groups. Simple-effect LSD-t for pairwise comparison revealed that there were significant differences in MVV%, FVC% and FEV1% between the two groups at different time points (p<0.05). While there was no significant difference in MVV%, FVC% and FEV1% between the two groups before operation (p>0.05). Meanwhile, at one month, three months and six months after the operation, MVV%, FVC% and FEV1% in the segmentectomy group were significantly higher than those in the lobectomy group, with statistically significant differences (p<0.05), as described in Table-III. Graphpad Prism was used to plot and analyze the pulmonary function indexes of the two groups, as shown in Fig.1-2.

**Table-III:** Comparison of pulmonary function indexes between the two groups (*χ̅*±*S*).

Indexes	Groups	*n*	Time point 1	Time point 2	Time point 3	Time point 4	F Time point	F Group	F interaction

			Before operation	1 month after operation	3 months after operation	6 months after operation			
MVV%	Segmentectomy group	40	96.32±9.54	91.08±8.75*	84.39±6.83*	80.12±5.07*	218.643	17.198	8.846
	Lobectomy group	40	95.18±9.26	86.54±8.87*	77.61±6.19*	70.55±5.23*			
	t		0.542	2.305	4.652	8.310	-	-	-
	P		0.598	0.024	<0.001	<0.001	<0.001	<0.001	<0.001
FVC%	Segmentectomy group	40	95.72±9.68	91.80±8.92*	87.04±8.31*	84.17±7.64*	123.804	12.094	8.573
	Lobectomy group	40	95.26±9.35	86.59±8.43*	80.11±7.79*	75.23±6.87*			
	t		0.216	2.685	3.848	5.503	-	-	-
	P		0.829	0.009	<0.001	<0.001	<0.001	0.001	<0.001
FEV1%	Segmentectomy group	40	95.49±8.85	90.50±8.12*	87.35±7.84*	83.28±7.01*	139.026	24.471	14.956
	Lobectomy group	40	94.82±8.37	85.33±7.61*	78.21±7.32*	71.19±6.30*			
	t		0.348	2.938	5.389	8.113	-	-	-
	P		0.729	0.004	<0.001	<0.001	<0.001	<0.001	<0.001

***Note:*** Compared with the level before operation within groups,

* p<0.05.

As for the degree of postoperative pain, the results of Mauchly’s Test of Sphericity showed that the VAS scores of the two groups met the Huynh-Leldt condition (p>0.05). Repeated measurement analysis revealed significant differences in intra-subject and inter-subject effects of VAS score between the two groups (p<0.05), suggesting that VAS varied with time and corresponding changes were different between groups. Pairwise comparison with simple-effect LSD-t (Table-IV) showed that there were significant differences in VAS score of the two groups at different time points (p<0.05); and the segmentectomy group had evidently lower VAS scores at one day, three days and five days after the operation than those in the lobectomy group, with statistically significant difference (p<0.05). Fig.2 presents the change trend of VAS scores of the two groups over time.

**Table-IV:** Comparison of the degree of postoperative pain between the two groups (*χ̅*±*S*).

Index	Groups	*n*	Time point 1	Time point 2	Time point 3	F Time point	F Group	F interaction

			1 d after operation	3 d after operation	5 d after operation			
VAS score	Segmentectomy group	40	5.78±1.13	3.58±0.96*	2.31±0.72*	329.253	60.972	17.168
	Lobectomy group	40	6.61±1.24	5.02±1.10*	4.45±0.89*			
	t		-3.129	-6.241	-11.821	-	-	-
	P		0.024	<0.001	<0.001	<0.001	<0.001	<0.001

***Note:*** Compared with the score 1d after operation within groups,

* p<0.05.

Comparison of complications between the two groups (Table-V) indicated a statistically significant difference that the total incidence of complications was significantly lower in the segmentectomy group than that in the lobectomy group (7.50% vs 25.00%) (p<0.05).

**Table-V:** Comparison of complications between the two groups (n, %).

Groups	n	Pulmonary infection	Pulmonary leakage	Intestinal obstruction	Arrhythmia	Atelectasis	Total incidence
Segmentectomy group	40	1 (2.50)	0 (0.00)	1 (2.50)	1 (2.50)	0 (0.00)	3 (7.50)
Lobectomy group	40	2 (5.00)	1 (2.50)	4 (10.00)	2 (5.00)	1 (2.50)	10 (25.00)
*χ* ^2^	-	-					4.501
*P*	-	-					0.034

## DISCUSSION

In our study, despite the presence of pulmonary function damage in both groups, the segmentectomy group showed significantly improved MVV%, FVC% and FEV1% than those in the lobectomy group at one month, three months and six months after the operation. Moreover, the VAS score of the segmentectomy group was significantly lower than that of the lobectomy group at first, third and fifty day after the operation. It is suggested that segmentectomy may have less damage to the pulmonary function in the treatment of stage I NSCLC. In terms of the possible reason, thoracoscopic segmentectomy can effectively reduce the damage to pulmonary function and alleviate the pain by preserving the lung volume effectively. In addition, the total incidence of complications were remarkably lower in the segmentectomy group than that in the lobectomy group (7.50% vs 25.00%). Clearly, the application of thoracoscopic segmentectomy can reduce complications and has high safety, which can be explained by the smaller incision and reduced incidence of postoperative infection.^8^,^9^

NSCLC is a lung malignant tumor with a relatively localized lesion location that accounts for about 4/5 of all lung cancers, which is characterized by less tumor metastasis and local thoracic infiltration.^10^-^12^ The cure rate is relatively high for early NSCLC patients undergoing surgical resection. Traditionally, early NSCLC can be treated by open lobectomy through the removal of the diseased lobes to dissect the lymph nodes, completely remove the lesions and improve the long-term survival of patients eventually; which, however, may have a serious impact on the pulmonary function of patients that is not conducive to subsequent recovery.^13^,^14^ Therefore, it is of great importance for postoperative recovery to explore a safe and effective surgical approach for the treatment of stage I NSCLC.

Thoracoscopic lobectomy is gradually applied to early NSCLC with the development of medical technology. It has been widely recognized by doctors and patients owing to its advantages of minimally invasive, less pain, less intraoperative blood loss and rapid postoperative recovery.^15^,^16^ However, NSCLC occurs primarily in the elderly who have multiple underlying diseases, decreased immunity, degraded physiological function and reduced lung compliance. There may be a decreased tolerance of patients with thoracoscopic lobectomy accordingly, resulting in a significant decline in postoperative pulmonary function and an adverse impact on the quality of life.^17^

In recent decades, segmentectomy is highly valued in the field of medicine. Acting as an anatomical resection, it can realize the dissection and separate treatment of blood vessels and bronchus of the pulmonary segments that need to be removed without using the spreader and expanding the incision, showing good clinical therapeutic effect and high safety.^18^ However, compared with thoracoscopic lobectomy, thoracoscopic segmentectomy is more complicated in procedure and has strict technical requirements for operation; besides, it has more complex dissection and prolonged duration of operation owing to the deep location of pulmonary bronchi and blood vessels.^19^ In this study, a similar result was observed that the segmentectomy group had obviously a longer duration of operation than that of the lobectomy group. The segmentectomy group also showed significantly less thoracic drainage volume and shorter length of stay in hospital stay than those in the lobectomy group. These data support that patients undergoing segmentectomy have relatively less pulmonary tissue exudation and more rapid postoperative recovery.

In the case of a small range of lesions, the lung tissue has a strong compensatory capacity to supplement the damaged ventilation by redistributing the lung tissue, without an obvious impact on the pulmonary function.^20^

### Limitations of this study:

It includes a small number of patients were included and no long-term follow-up was conducted. In view of this, more samples should be included and follow-up time should be increased in future studies to further validate the findings of this study.

## CONCLUSIONS

Thoracoscopic segmentectomy is more effective in the treatment of stage I NSCLC than lobectomy, with less bleeding and mild pain, which can alleviate pulmonary function injury and reduce postoperative complications that is conducive to the improved prognosis of patients. However, it proposes a higher requirement for operators due to its more complicated procedure and longer duration of the operation.

### Authors’ Contributions:

**HJ** and **BZ:** Designed this study, prepared this manuscript, are responsible and accountable for the accuracy or integrity of the work.

**TW** and **HW:** Collected and analyzed clinical data.

**PQ:** Performed the statistical analysis and participated in its design.
